# Clinically Relevant Prenatal Ultrasound Diagnosis of Umbilical Cord Pathology

**DOI:** 10.3390/diagnostics12020236

**Published:** 2022-01-19

**Authors:** Roxana Elena Bohîlțea, Vlad Dima, Ioniță Ducu, Ana Maria Iordache, Bianca Margareta Mihai, Octavian Munteanu, Corina Grigoriu, Alina Veduță, Dimitrie Pelinescu-Onciul, Radu Vlădăreanu

**Affiliations:** 1 Department of Obstetrics and Gynecology, “Carol Davila” University of Medicine and Pharmacy Bucharest, 37 Dionisie Lupu, 020021 Bucharest, Romania; dimitriepelinescu@yahoo.com (D.P.-O.); vladareanu@gmail.com (R.V.); 2 Department of Obstetrics, Gynecology and Neonatology, Filantropia Hospital, 11–13 Ion Mihalache Blv., Sector 1, 011171 Bucharest, Romania; bmmihai@gmail.com (B.M.M.); alina.veduta@gmail.com (A.V.); 3 Department of Obstetrics and Gynecology, University Emergency Hospital, 169 Splaiul Independentei Bld., Sector 5, 050098 Bucharest, Romania; ionitaducu@gmail.com; 4 Optospintronics Department, National Institute for Research and Development in Optoelectronics-INOE 2000, 409 Atomistilor, 077125 Magurele, Romania; 5 Department of Anatomy, “Carol Davila” University of Medicine and Pharmacy Bucharest, 37 Dionisie Lupu, 020021 Bucharest, Romania; octav_munteanu@yahoo.com

**Keywords:** umbilical cord, prenatal diagnosis, ultrasound, 3D imaging, color Doppler, vasa praevia, velamentous cord insertion, umbilical knot, second trimester screening

## Abstract

Umbilical cord abnormalities are not rare, and are often associated with structural or chromosomal abnormalities, fetal intrauterine growth restriction, and poor pregnancy outcomes; the latter can be a result of prematurity, placentation deficiency or, implicitly, an increased index of cesarean delivery due to the presence of fetal distress, higher admission to neonatal intensive care, and increased prenatal mortality rates. Even if the incidence of velamentous insertion, vasa praevia and umbilical knots is low, these pathologies increase the fetal morbidity and mortality prenatally and intrapartum. There is a vast heterogeneity among societies’ guidelines regarding the umbilical cord examination. We consider the mandatory introduction of placental cord insertion examination in the first and second trimester to practice guidelines for fetal ultrasound scans. Moreover, during the mid-trimester scan, we recommend a transvaginal ultrasound and color Doppler assessment of the internal cervical os for low-lying placentas, marginal or velamentous cord insertion, and the evaluation of umbilical cord entanglement between the insertion sites whenever it is incidentally found. Based on the pathological description and the neonatal outcome reported for each entity, we conclude our descriptive review by establishing a new, clinically relevant classification of these umbilical cord anomalies.

## 1. Introduction

During embryogenesis, the four folds that emerge on the surface of the embryonic disc in the fourth embryonic week converge centrally in the umbilical area. The primitive umbilical ring is an oval reflection line between the amnion and embryonic ectoderm, which in the 5th week of the embryonic stage, passes the yolk duct and allantois together with the yolk vessels. The embryonic allantoid arteries form the umbilical arteries, and from the left allantoid vein, the umbilical vein is formed. The right umbilical vein that usually degenerates may persist as the single umbilical vein or the fourth vessel of the cord. The connection pedicle that consists of the allantois and umbilical vessels (two arteries and a vein), along with the yolk pedicle, passes through the canal that connects the intraembryonic and the extraembryonic cavities. Subsequently, being covered by the amnion, the pedicles form the primitive umbilical cord [1]. The rapid growth of the intestine and liver in the 6th week of embryologic development causes a temporary inadequacy of the abdominal cavity for its contents; as a repercussion, there is an intestinal protrusion in the extraembryonic residual coelom, at the base of the umbilical cord. At the end of the first trimester, the yolk sac located in the chorionic cavity degenerates; thus, physiological herniated intestinal loops resolve after 12 weeks of gestation [2,3]. Subsequently, the umbilical cord vessels remain just surrounded by Wharton’s jelly, a mucoid matrix of mesodermal origin. Umbilical arteries present a helical disposition around the vein, forming tortuosities and false nodes.

Umbilical cord anomalies (UCA) are not rare and are often associated with structural or chromosomal abnormalities, fetal intrauterine growth restriction, and poor pregnancy outcomes due to prematurity and placentation deficiency. The NICHD Stillbirth Collaborative Research Network Group reported in 2020 that, from 496 stillbirths wherein cause of death was analyzed and documented using the INCODE (Initial Causes of Fetal Death) classification system, 19% of deaths were due to umbilical cord abnormalities: a total of 27% by umbilical cord knots, torsions or stricture; 29% by nuchal, shoulder or body umbilical cord entanglement; and 5% were complicated by umbilical cord prolapse [4].

The International Society of Ultrasound in Obstetrics and Gynecology (ISUOG) practice guidelines do not recommend screening for umbilical cord abnormalities. According to its 2013 Practice Guidelines for first-trimester fetal ultrasound scans, the numbers of cord vessels, fetal insertion of the umbilical cord, and the presence of cord cysts should be documented [5]. Imaging the umbilical cord during second trimester prenatal ultrasound examination is optional and limited to determining the number of vessels in the cord and assessment of the fetal insertion site; the study of placental insertion is proposed only for multiple gestations, even if the association of this pathology with pregnancy complications is recognized [6]. ISUOG Practice Guidelines: ultrasound assessment of fetal biometry and growth, from 2019 [7], mentions that future areas of research should include functional imaging of the placenta, which could improve perinatal outcomes. In the last two years, the guidelines have been updated to include Doppler ultrasonography in feto–placental circulation [8,9] and the role of ultrasound in congenital infection [10]; these guidelines stipulate the need for placental cord insertion in preparation for ultrasound-guided invasive procedures [11].

The American Institute of Ultrasound in Medicine (AIUM) guidelines for obstetric ultrasound examination sustain the necessity of imaging the umbilical cord to establish the number of vessels, and the fetal and placental insertion sites, for the standard second- and third-trimester ultrasound examination [12]. The Australasian Society for Ultrasound in Medicine recommends a placental cord insertion evaluation, specifying the variety of marginal and velamentous anomalies; the guideline suggests the benefit of transvaginal color or power Doppler scans with the intent of ruling out vasa praevia [13]. The American College of Radiology (ACR) and the American College of Obstetricians and Gynecologists (ACOG) do not recommend routine evaluation of the placental insertion of the cord, but recommend, when possible, imaging the umbilical cord and the number of vessels [14]. The Society of Obstetricians and Gynaecologists of Canada recommends placental cord insertion evaluation only in cases of low-lying placenta, also advising for transvaginal evaluation of the internal cervical os in cases of placenta praevia, low or velamentous insertion of the cord, vaginal bleeding, bilobed, or succenturiate placenta [15]. The Royal College of Obstetricians and Gynecologists considers that there are insufficient arguments for general population second trimester screening for vasa praevia, even though a transvaginal ultrasound scan presents elevated accuracy, as well as a decreased false positive rate [16]. However, all other pathologies of the umbilical cord (Table 1) are only considered as incidental findings and are not specifically screened for. The evaluation of the free cord loops that could reveal true knots, position, structure and helical pattern anomalies, is not stipulated in any guide.

Both normal anatomy and malformations can be depicted by conventional 2D imaging, but color Doppler should be routinely used for umbilical cord assessment, especially in the second half of pregnancy; the benefit of 3D imaging techniques in the diagnosis of umbilical cord knot is indisputable, and enhanced by the HD-flow mode; however, the key to diagnosis is searching for the anomaly [17]. Among the advantages of 3D imaging in the assessment of fetal abnormalities, the most important one refers to the availability of several display modes, which allow the demonstration of even subtle fetal defects from an optimal angle [18]. Compared to 2D ultrasonography, 3D imaging can assess up to 60.8% of the antenatal defects, proving to be a useful tool in appreciating the severity of a fetal defect [19]. This is especially important when counseling the parents, since virtual examination can help them “see” the severity of a malformation, or the absence of any fetal abnormality [18,20].

The two arteries and the vein composing the umbilical cord should be assessed in both the transverse and longitudinal planes, always associating the color Doppler ultrasound image of the paravesical umbilical vessels in the axial view of the fetal pelvis, in order to exclude single umbilical artery variants. Transvaginal ultrasound is recommended for high-risk pregnancies with placentation anomalies where vasa praevia is suspected [13,21]. Sonographically, the umbilical cord can be visualized starting from eight weeks of gestation. From the 10th week of gestation it is possible to determine the number of vessels trough their visualization in the paravesical section using the color Doppler technique. Combined use of transabdominal and transvaginal ultrasound permits a better diagnosis of the placental type, situation, and cord insertion.

Extended analysis of the umbilical cord insertions and tract might offer the advantage of identifying and preventing adverse perinatal outcomes associated with certain umbilical cord abnormalities.

## 2. Ultrasonographic Aspects of Pathological Classification of UCA

In Table 1, we present a summary of the general classification of UCA, indicating the risk factors, incidence, associated pathology and the outcome.

**Table 1:** General classification of UCA.

	Pathology		Incidence	Risk Factors	Associated Pathology	Adverse Outcome	Reference
Placental Insertion Anomalies	Velamentous Cord Insertion		0.23–1% singleton gestation 15% monochorionic twin pregnancy	▪ ART (IVF), low lying placenta, placenta praevia ▪ Accessory lobe or bilobated placenta ▪ Multiple pregnancy	▪ Vasa praevia ▪ Single umbilical artery—12.5% of cases ▪ Fetal anomalies	Intrauterine growth restriction, preterm labor, placental abruption, low Apgar score, intrauterine fetal death, acute fetal distress by rupture, kinking, or compression of the cord insertion, hemorrhage and obstetrical maneuvers for placental retention in the third stage of labor, vessel thrombosis with placental infarction and distal segment fetal amputation, neonatal purpura, twin–twin transfusion syndrome	[14,22,23,24,25,26,27,28,29]
	Vasa praevia		0.0004–0.08%	▪ Second-trimester low-lying placenta or placenta praevia, bilobed or succenturiate lobe placenta ▪ Assisted reproductive technologies ▪ Velamentous cord insertion ▪ Vaginal bleeding ▪ Multiple gestation ▪ First trimester umbilical cord insertion in the lower 1/3 of the uterus	▪ Fetal heart abnormalities	Preterm birth (<32 weeks), SGA neonate, neonatal death, postpartum hemor-rhage, emergency cesarean section, elective cesarean section, admission to NICU, neonatal blood transfusion	[16,25,30,31]
	Eccentric/Marginal Cord Insertion		7% singleton pregnancy 25% twin pregnancy	Advanced maternal age (≥35 years) Chronic maternal pathologies Female fetus Marginal cord insertion in previous pregnancy		Intrauterine growth restriction, preterm labor, progression to velamentous cord insertion, high risk of cesarean section, low Apgar score Fetal malformations, NICU admission, preeclampsia, emergency and elective cesarean section	[27,31,32,33]
Fetal Insertion Anomalies	Omphalocele and gastroschisis		0.08%	▪ Extreme ages (under 20 and over 40 years) ▪ Maternal obesity ▪ Inconstantly demonstrated teratogenicity caused by selective serotonin reuptake inhibitors	▪ Fetal aneuploidies, ▪ Gastrointestinal abnormalities ▪ Cardiac defects ▪ Genitourinary, orofacial and diaphragmic malformations ▪ Neural tube defects ▪ Polyhydramnios ▪ Cantrell pentalogy, ▪ Amniotic bridle sequence ▪ Fusion defect association ▪ OEIS syndrome, Shprintzen syndrome, Carpenter syndrome, Goltz syndrome, Meckel–Gruber syndrome, CHARGE syndrome and Beckwith–Wiedemann syndrome	Intrauterine growth restriction, prematurity, elective cesarean section	[34,35,36,37,38,39,40]
Positional anomalies Cord anomalies		Nuchal Cord	Between 35% and 0.6% (>3 loops)	▪ Excessive fetal movement ▪ Excessive long umbilical cord ▪ Monoamniotic twins ▪ Number of loops increases with gestational age ▪ Male fetuses	Cord knot	Intrauterine growth restriction, acute fetal distress, perinatal death, stillbirth, operative vaginal delivery, emergency cesarean delivery, need of oxygen supplementation at delivery	[41,42,43,44,45,46,47]
		Cord Knot	0.3–1.3%	▪ Advanced maternal age ▪ Multiparity ▪ Obesity ▪ Previous spontaneous abortion ▪ Chronic hypertension ▪ Gestational diabetes	Long umbilical cord length	Prematurity, low Apgar score, NICU admission, emergency cesarean delivery, elective cesarean delivery, antepartum and intrapartum fetal death (likelihood of stillbirth is more than 4-fold higher)	[17,48,49]
		Cord Strictures	rare	Twin pregnancy	▪ Umbilical cord overcoiling ▪ Long umbilical cord length	Intrauterine growth restriction, Intrauterine fetal death	[50]
	Structural anomalies	Single Umbilical Artery	0.55–5.9%	▪ Extremes of maternal age ▪ Diabetes ▪ Smoking ▪ Hypertension ▪ Twin pregnancy	▪ Genitourinary malformations ▪ Caudal regression syndrome ▪ Sirenomelia ▪ Cardiac anomalies ▪ Gastrointestinal anomalies ▪ Musculoskeletal anomalies ▪ Central nervous system anomalies	High rate of pregnancy loss Intrauterine growth restriction Iatrogenic prematurity	[51,52,53,54,55,56,57]
		Umbilical artery hypoplasia	0.04%	Maternal diabetes mellitus	▪ Placentation anomalies ▪ Abnormal placental cord insertion ▪ Trisomy 18 ▪ Agenesis of corpus callosum ▪ Cardiac anomalies ▪ Genitourinary minor malformations ▪ Polyhydramnios	Intrauterine growth restriction	[58]
		Supernumerary vessels (Right Umbilical Vein Persistence)	0.5%	▪ Twin pregnancy (thoracopagus and omphalopagus twins) ▪ Thrombus obstruction, teratogens or folic acid deficiency ▪ Female fetuses	▪ Anterior chest wall defects ▪ Bilateral cleft lip and palate ▪ Placental arteriovenous fistula ▪ Edema ▪ Heterotaxy syndrome ▪ Trisomy 18 ▪ Holoprosencephaly ▪ Polyhydramnios ▪ Omphalocele ▪ Triploidy ▪ Hypertrophic cardiomyopathy ▪ Ectopia cordis ▪ Tetralogy of Fallot ▪ Ductus venosus agenezia (DV)	Intrauterine growth restriction	[59,60,61,62]
		Umbilical Cord Cyst	2–3%	Chromosomal anomalies	▪ Fetal aneuploidies ▪ Omphalocele ▪ Vertebral defects ▪ Imperforate anus ▪ Tracheoesophageal fistula ▪ Radial and renal dysplasia association ▪ Angiomyxoma of the cord	Rapid enlargement with the restriction of blood flow and fetal distress requiring emergency birth. Torsion or thrombosis may cause fetal demise	[63]
		Cord Hematoma	9 × 10^−5^	▪ Umbilical blood sampling ▪ Fetal transfusion	Fetal bradycardia	Modified umbilical artery flow velocimetry, perinatal hypoxia, miscarriage	[64,65]
		Cord Varix/Aneurysm	0.0011%	▪ Chromosomal anomalies ▪ Single umbilical artery ▪ Male fetus	▪ Chromosomal anomalies ▪ Anatomical abnormalities ▪ Single umbilical artery	Intrauterine death by aneurysm rupture or varix thrombosis, fetal hydrops, SGA, invalidated neurodevelopmental delay	[61,66,67]
		Cord Tumors: angiomixomas, mixosarcomas, coriomixomas, hemangiomas, teratomas	Isolated cases	Twin pregnancy for teratomas	Teratomas assoaciate: ▪ Omphalocele ▪ Trisomy 13 Angiomyxoma assoaciate: ▪ Skin hemangiomas	Intrauterine death due to torsion or compression effect on umbilical cord vessels	[68,69]
	Coiling and length anomalies	Excessive/Absent Coiling	4–5%	Abnormal placentation	Single umbilical artery	Fetal growth restriction, congenital anomalies, fetal heart rate abnormalities, preterm birth, intrauterine death	[70]
		Abnormally short/long Cord	8.26%	Fetal malformations	▪ Fetal malformations ▪ Myopathic and neuropathic diseases	Fetal inactivity in cases of short umbilical cord, oligohydramnios, placental pathology, fetal growth restriction, long cord entanglement and intrauterine asphyxia and fetal death.	[71,72,73]

### 2.1. Velamentous Cord Insertion

**Figure 1:**
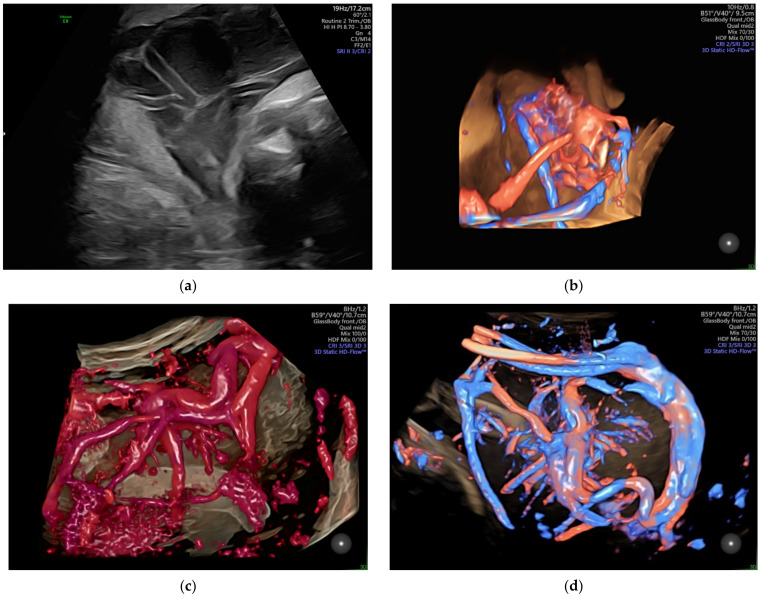
Velamentous cord insertion as (**a**) 2D scan and (**b**–**d**) 3D Static HD Flow imaging.

The velamentous insertion of the umbilical cord (Figure 1) characterized by the divergence of the umbilical vessels as they traverse the amnion and the chorion before reaching the placenta, associates important obstetrical complications; the diagnosis by ultrasonography as early as possible is important for the guidance of the subsequent management.

Typically, the umbilical vessels lie parallel to the uterine wall as they penetrate the placental pole. Velamentous cord insertion is one of the most undiagnosed conditions in obstetrics. The velamentous cord insertion can be diagnosed by ultrasound, with a sensitivity of 69% to 100% and a specificity of 95% to 100%, in the second trimester [24]. In the third trimester, this condition is also reflected through variable decelerations and abnormal fetal heart rate variability in a non-stress test; this is frequently associated with vasa praevia, the most reliable method of diagnosis for which is the real-time color Doppler transvaginal ultrasound examination, which can depict the umbilical vessel pathway, which crosses the internal os or passes at less than 2 cm from it; this is used to study the end-diastolic velocity of the umbilical artery. The single umbilical artery is associated with velamentous cord insertion in 12.5% of cases [23]. Our previous study, which included 43 cases of velamentous cord insertion from 18,500 deliveries in our department during a 6 year period, reported an incidence of associated single artery umbilical cord of 27.9% [25,26].

### 2.2. Vasa Praevia

**Figure 2:**
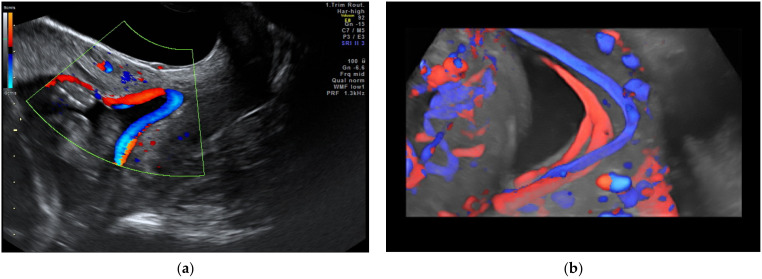
Vasa praevia as (**a**) 2D color Doppler ultrasound image and (**b**) 3D Static HD Flow imaging.

Vasa praevia (Figure 2) are the umbilical vessels that cross the membranes of the lower uterine segment. The main risk associated with this abnormality is the rupture of vessels, even without rupture of the membranes resulting in fetal exsanguination or compression by the fetal presentation part. The antenatal diagnosis of vasa praevia increases the neonatal survival from 44% to 97% and improves the neonatal outcome [24,74]. Management particularities include a non-stress examination twice a week after 28 weeks of gestation, and cesarean delivery between 34–36 weeks of gestation [30].

Three types of vasa praevia are described: type 1, resulting from velamentous cord insertion; type 2, presented in patients with bilobed or succenturiate lobed placenta; and type 3, boomerang-shaped vessels that cross the membranes along the placental margin, similar to resolving placenta praevia [30,75,76]. Antenatal diagnosis is based on transvaginal ultrasound examination and color Doppler flow mapping that can highlight umbilical vessels located 2 cm proximal to the cervical os [26,76]. Two-stage screening for vasa praevia, based on transvaginal sonography at 20–22 weeks of gestation, has recently been proposed by Zhang W et al. regarding pregnancies with velamentous cord insertion at the routine 11–13-week scan, and those with low-lying placenta at the mid-trimester scan; according to the authors, this method of screening would diminish stillbirths by 10% [31].

### 2.3. Marginal Cord Insertion

**Figure 3:**
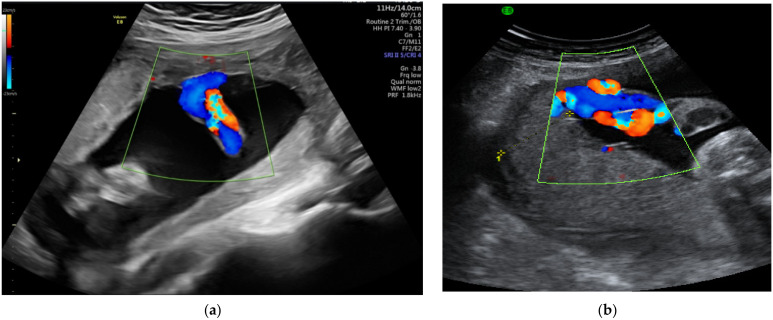
(**a**,**b**) Marginal insertion of the umbilical cord seen by color Doppler ultrasound.

The defining element in describing marginal insertion of the umbilical cord (Figure 3) is for the cord insertion to be located within 2 or 3 cm of the placental edge [77,78,79]. Although many authors consider that there is no increased risk, complications such as intrauterine growth restriction, preeclampsia, preterm labor and progression to velamentous cord insertion were described [32,78]. According to Zhang W et al. [31], 9.5% of cases diagnosed with vasa praevia during the second trimester scan presented marginal insertion of the umbilical cord during the first trimester screening.

### 2.4. Anomalies of the Fetal Abdominal Cord Insertion

**Figure 4:**
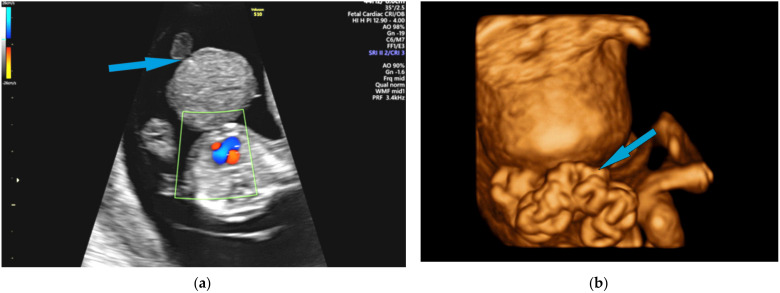
(**a**) Omphalocele and (**b**) gastroschizis ultrasound images of the 13^+^ gestational week–old fetuses on 2D color Doppler and 3D reconstruction, respectively; the blue arrow indicates fetal umbilical cord insertion.

Omphalocele and gastroschisis are the most common anterior abdominal wall defects (Figure 4). Prenatal ultrasound diagnosis of the non-liver-containing omphalocele is certain after 12 weeks of gestational age, whereas extrabdominal liver tissue could be observed transvaginally at 9–10 weeks of amenorrhea [80]. The omphalocele with intestinal content associates, in 60% of cases, with aneuploidies; as it can be part of a series of syndromes, it is mandatory that ultrasound assessment carefully looks for associated structural anomalies. Regarding umbilical cord insertion, the ultrasound differential diagnosis mainly targets gastroschisis in the presence of an unaffected insertion, adjacent to the abdominal wall defect.

### 2.5. Single Umbilical Artery

There are four types of single umbilical artery: type 1, which corresponds to 98% of cases, and is characterized by the presence of an artery of allantoic origin and a normal vein. The association of the type 1 single umbilical artery with genitourinary anomalies is frequently described; type 2, which corresponds to about 1.5% of cases, and is characterized by the presence of an artery of vitelline origins and a normal vein. The association with caudal regression syndrome and sirenomelia was described for this type; type 3 and 4, which are characterized by their rarity, consisting of two veins and one artery of allantoic origin in type 3, and one persistent anomalous right umbilical vein and one artery in type 4, respectively. For these two types, the association of severe fetal anomalies with poor fetal prognosis is common; moreover, the risk of pregnancy loss is high [81].

**Figure 5:**
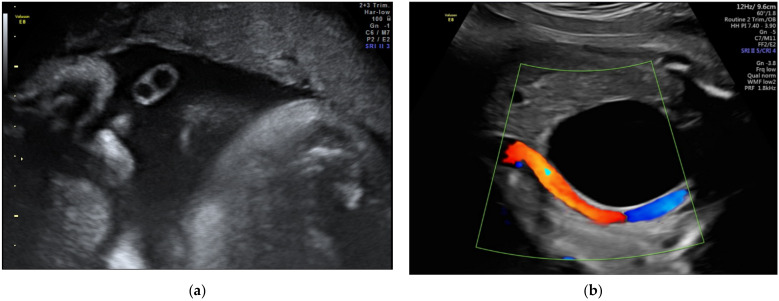
Single umbilical artery.(**a**) 2D imaging and (**b**) color Doppler imaging.

Single umbilical artery can be diagnosed in the first trimester, but confirmation in the second trimester, by visualizing the intra-abdominal pathognomonic umbilical vessel present on only one side of the fetal bladder, is mandatory. The cord may also be velamentously inserted and thin (Figure 5) [82,83]. Along with the confirmed diagnosis, a special assessment of the possible associated anomalies (genitourinary, cardiac, gastrointestinal, musculoskeletal, central nervous system) is required [84]. The results of a large study that evaluated the associated conditions of single umbilical artery showed a higher prevalence of cardiac and renal anomalies in this group [85]. Additional invasive genetic studies have no justification in isolated cases of single umbilical artery, since the rate of aneuploidy among these cases is not increased; however, in cases of suspected additional fetal abnormalities, genetic invasive testing with karyotyping and microarray is recommended [86]. Regarding the management particularities, The Society of Obstetricians and Gynecologists of Canada recommendations include follow-up physical and sonographic evaluations of an eventual fetal growth restriction [87].

### 2.6. Umbilical Artery Hypoplasia

**Figure 6:**
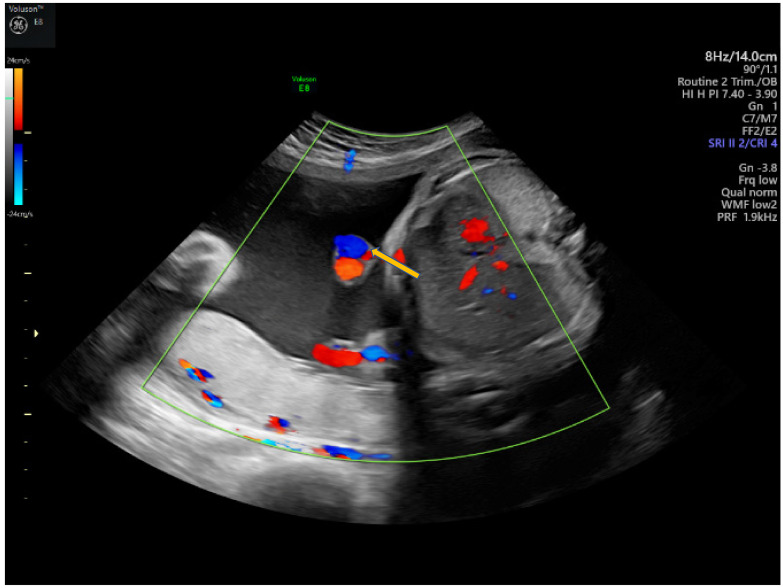
Hypoplasia of the umbilical artery (arrow).

Umbilical artery hypoplasia (Figure 6) is defined by an artery caliber difference of over 50% and by the associated discordant umbilical artery-flow velocity with the resistance index almost always increased, or an absent end-diastolic flow in the smaller artery [58]. The clinical significance of this anomaly is not clearly established yet, but it seems to be associated with placental anomalies and abnormal placental cord insertion, trisomy 18, agenesis of corpus callosum, and cardiac and genitourinary minor malformations. Since umbilical artery hypoplasia could be a risk factor for fetal growth restriction, careful fetal anatomy and growing surveys are recommended.

### 2.7. Supernumerary Vessels

**Figure 7:**
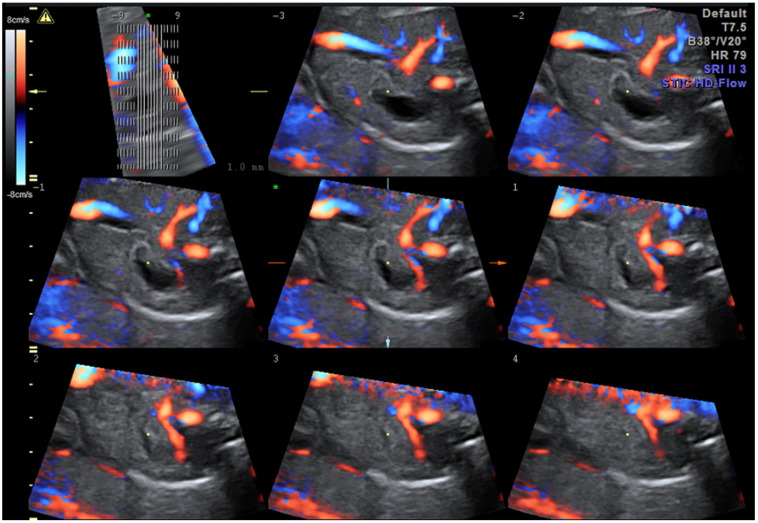
Two-dimensional imaging of a double umbilical vein.

The typical model of multivessel cord most commonly encountered contains two arteries and two veins (Figure 7). A result of persistence of the right umbilical vein or of an abnormal splitting of an umbilical vessel, four-vessel umbilical cord is a common finding in conjoined twins and a very rare presence in singletons; this occurrence is usually associated with multiple and severe congenital anomalies, thus, pregnancy management depends on the associated findings [59,60]. The prenatal diagnosis of persistent right umbilical vein requires venous system scanning in 2D color-Doppler mapping; the transverse and longitudinal sections of the fetal abdomen reveal an abnormal course of the portal vein toward the stomach, the umbilical vein located lateral to the fetal gallbladder, and curving of the connection of the umbilical vein to the portal vessels towards the stomach [62].

### 2.8. Cord Knots

**Figure 8:**
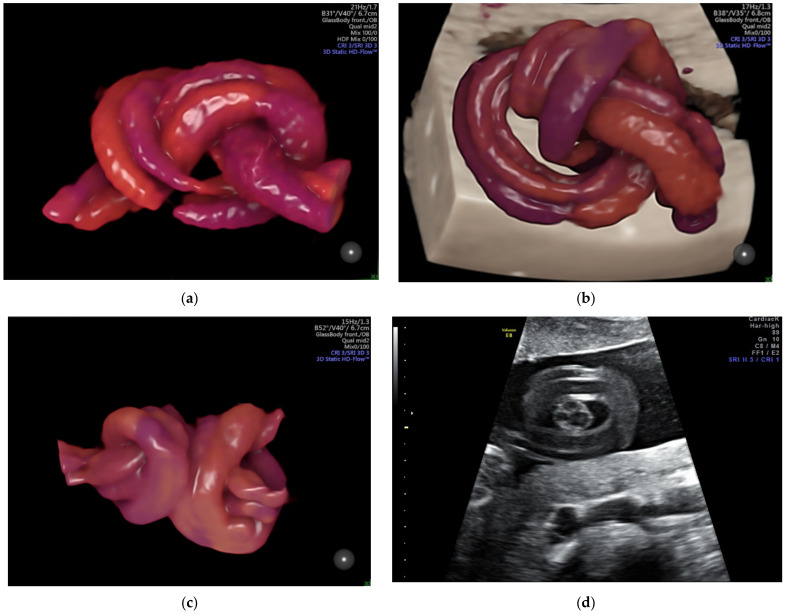
Images of true umbilical knots as (**a**–**c**) 3D Static HD Flow imaging and (**d**) 2D ultrasound image.

A true umbilical cord knot (Figure 8 and Video S1) often remains undiscovered prenatally, the ultrasound visualization of the entanglement being an incidental finding. It is unusual for a knot to tighten, especially before the onset of labor. The prenatal suspicion of the presence of a true umbilical cord knot appears when a cross-section of an umbilical cord surrounded by a circular loop is observed on gray-scale ultrasound [88]; ultrasound achievements in 3D and Doppler mode easily sustain prenatal positive diagnosis, and distinguish from the false knots, which are more often suggested by the four-leaf clover appearance [17]. The specificity of the diagnosis is increased if the image persists even after the fetus has changed his position, and the same image is captured in two subsequent examinations. Unfortunately, the features are almost identical to the false knot, representing arterial vascular loops formed helically around the umbilical vein; therefore, confirmation and differential diagnosis requires the acquisition of three-dimensional volume color-Doppler of the suspected anatomical section. The compression of the cord by a constricted true knot can be detected by pulsed Doppler velocimetry of the umbilical artery [89]. The impact on the neonatal outcome of a prenatal diagnosis of a true umbilical cord knot has not been fully evaluated [49]; a recent publication noted that cord entanglement does not contribute to prenatal morbidity and mortality in monoamniotic twin pregnancies [41].

We have revealed, in a previous study [17], an umbilical cord knot incidence of 0.71%, with only 12% of them being antepartum diagnosed by ultrasonography; a false knot was recorded in 0.02% of cases. Most cases of true umbilical cord knots were diagnosed in women in their second and third pregnancies, at 42.8% and 30%, respectively. None of the patients with antepartum diagnosis of umbilical knot accepted vaginal delivery. Umbilical cord length was over 30% higher than the mean in these patients.

### 2.9. Nuchal Cord

**Figure 9:**
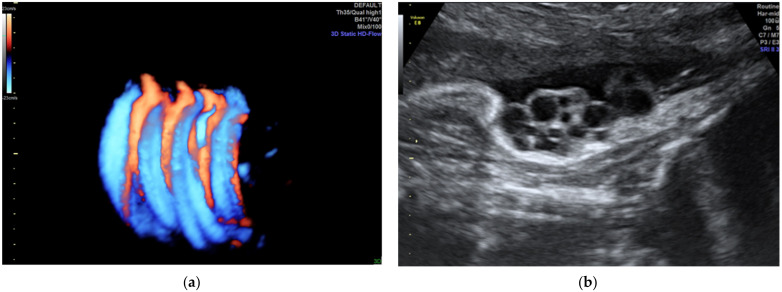
Example of a complex nuchal cord counting 5 loops (**a**) 3D Static HD Flow imaging and (**b**) 2D ultrasound image.

The actual impact of the nuchal cord (Figure 9 and Figure S1) in the pregnancy outcome is controversial. The number of loops is inversely correlated with incidence, and increases linearly with every week of gestation [42]; two or more loops growing exponentially is concerning. A classification regarding the locked and unlocked pattern was described in 1997, and is based on the position of the placental end of the umbilical cord in relation with the umbilical end [90]. Using gray-scale ultrasound imaging, the diagnosis sensitivity is about 70%; this is increased by color Doppler and tridimensional technology to about 97%, and leads to specificity reaching 96% [91,92]. The accuracy is high, but as Peregine concluded in his article [50] the ultrasound diagnosis of the nuchal cord will only be useful if we also include predictors of a complicated outcome, such as the divot sign, which represents indentation of the subcutaneous tissue, resulting from compression of the nuchal cord on the fetal neck, imaging in the longitudinal posterior plane [93]. Recently, Sherer DM et al. defined “complex umbilical cord entanglement” as cases of true knot of the umbilical cord, cases of ≥3 loops of nuchal cords, or any combination of a true knot and nuchal cord in singletons [92]. In monoamniotic twins and in fetuses with intrauterine growth restriction, the measurement of the flow velocity of the cord can dictate future management. None of the guidelines of prenatal ultrasound examination include routine screening for nuchal cords, since they are considered an incidental normal finding.

### 2.10. Cord Strictures

Stricture of the umbilical cord is an uncommon condition characterized by a sharp narrowing of the umbilical cord, usually associated with long umbilical cords or hypercoiling of the cord; an isolated stricture or multiple strictures can narrow the umbilical cord [94]. Intrauterine growth restriction and intrauterine fetal death has been repeatedly associated with this condition. An article published by French et al. in 2005 suggests a recurrence in pregnancies involving cord strictures [95]. The major pathological characteristics are the absence of the Warton’s jelly, stenosis, obliteration of cord vessels at the narrow segment, and intravascular cord thrombosis. The cause of umbilical stricture is unknown [50]. An accurate antenatal diagnosis of this umbilical cord anomaly is a challenge for most obstetricians; the Doppler flow velocimetry with modified values could be predictive; however, a sudden change in fetal activity can also require consideration.

### 2.11. Cord Hematoma

**Figure 10:**
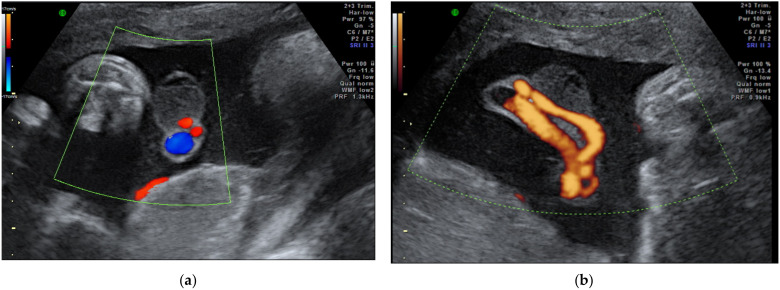
Cord hematoma seen on (**a**) 2D color Doppler imaging and (**b**) power Doppler imaging.

On prenatal ultrasounds, umbilical cord hematomas are seen as tumors (Figure 10 and Figure S2), presenting as a solid-appearing mass attached to, or within, the umbilical cord, but without the internal color spots of vessels. These pseudotumors, can occur spontaneously, but are more frequent a result of cordocentesis or fetal transfusion. Being seen on ultrasounds in close relation with the umbilical cord, hematomas can have different sizes and shapes depending on the appearance time. Acute hematomas are isoechoic/heterogeneous, while chronic hematomas are hypoechoic/anechoic. As a consequence, modified umbilical artery flow velocimetry can be observed due to the compression effect [64], along with fetal bradycardia. Usually an incidental finding, pregnancies with cord hematoma require special monitoring by blood flow velocity and non-stress tests twice weekly.

### 2.12. Cord Varix and Aneurysm

**Figure 11:**
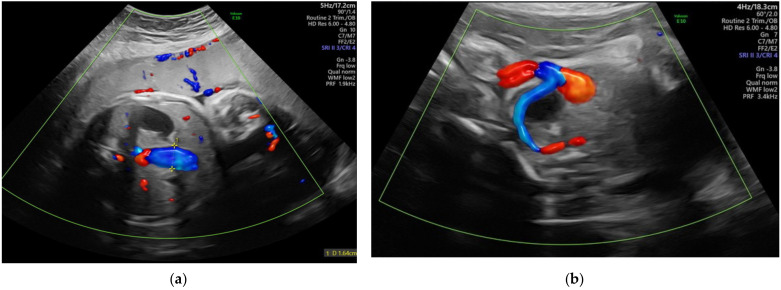
Two dimensional color-Doppler of umbilical vein varix typically occurring in the intraabdominal portion of the vein. (**a**) transverse abdominal section and (**b**) oblique vesical section.

Focal dilatation of the umbilical artery or vein are rare and have frequently been associated with fetal demise [96]. Upon prenatal ultrasound examination, cord varix can be seen as a cyst-like structure with venous flow on a Doppler examination. A value greater than 9 mm or 1.5-fold dilatation, compared to the normal adjacent segment of the umbilical cord, is required for the diagnosis [97]. The main complication is the thrombosis of the varix with hydrops fetalis as a direct consequence, but the recent multicenter cohort study of Novoa et al. sustains the association with chromosomal and/or anatomical abnormalities in 20% of cases [98]. Antenatal ultrasound reveals a cystic/elongated dilatation in close relation to the umbilical artery with non-pulsatile flow on a Doppler exam (Figure 11 and Figure S2).

### 2.13. Cystic Abnormalities—True Cysts and Pseudocysts

Upon ultrasound examination, both true and pseudocysts appear as hypoechoic lesion included in the umbilical cord, near the vessels. True cysts can be allantoically formed by the persistence of the urachus and are frequently associated with urachal anomalies and communication between the cyst and fetal bladder [99]; they can also be formed in an omphalomesenteric manner by the persistence of the vitelline duct, and present a difficult prenatal diagnosis [100]. The position of the allantoic cysts in the proximity of the fetal abdominal wall may be confounded with an anterior abdominal wall defect. In clinical practice, the risk related to this condition is rapid enlargement with the restriction of blood flow and fetal distress, requiring emergency birth. The particularities of the management of these cases include a weekly follow-up of umbilical artery velocimetry, and urological neonatal consultation.

**Figure 12:**
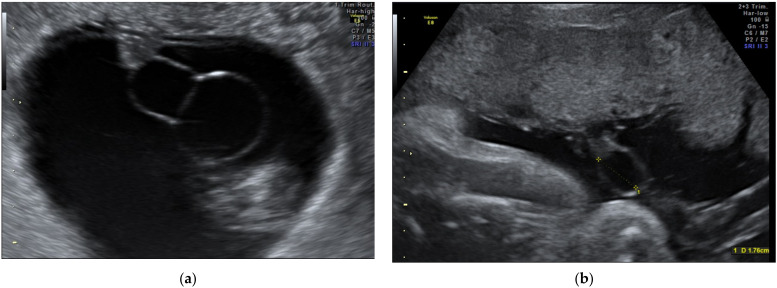
Cyst observed in the 2D ultrasound (**a**,**b**).

Umbilical cord pseudocysts are more common compared to true cysts (Figure 12 and Figure S3) [63]; they appear as a large, hypoechoic mass situated near to the fetal insertion or in a free loop of the cord, and compared with the yolk sac, present a less intense hypoechogenic wall, and are intra-amniotic. Differential diagnosis with an aneurismal umbilical vessel is made by color-Doppler. Regarding the strong association of pseudocysts with chromosomal abnormalities, their multiloculated aspect and their persistence over the 14th week must lead to prenatal karyotyping, detailed examination of fetal anatomy, and routine fetal growth evaluation [101]. Umbilical cord cysts diagnosed in the first trimester [63] usually have a rapid resolution, and their development is related with the cord coiling and formation of physiological midgut hernia. In case of large dimensions and severe fetal impact, aspiration of the cyst is indicated [102], but expectant management is sufficient in most cases.

### 2.14. Teratoma

Teratomas are rare heterogeneous tumors, that contain tissue from the three germ-cell layers, suspected to be small acardiac twins [68]. The cases detected prenatally in [103,104] were terminated, so the natural course of these pregnancies remained unknown. Differential diagnosis is made with placental teratoma that is located closely to the placental tissue or surrounded by it [69,105].

Angiomyxoma is an extremely rare tumor that presents as a hyperechogenic mass attached to the umbilical vessels; often associated with a pseudocyst, the blood vessels within the tumor have low flow velocities [106,107].

### 2.15. Coiling and Length Abnormalities

**Figure 13:**
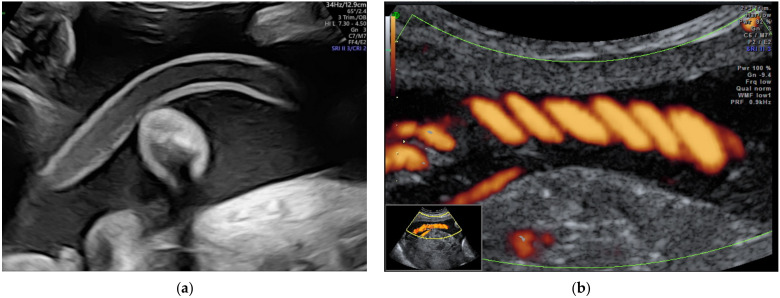
Coiling abnormalities: (**a**) lax cord and (**b**) hyperspiralized cord.

The role of coiling is to protect the cord from compression, kinking, and torsion, thus assuring an adequate blood supply to the fetus. Hypocoiled (Figure S4) or hypercoiled cord has also been associated with an increased rate of neonates that are small for their gestational age, congenital anomalies, fetal heart rate abnormalities, preterm birth and intrauterine death (Figure 13). Sonographic measurement of the number of complete coils per centimeter has an important degree of difficulty, and there are no prenatal, gestational-age-specific standard reference values [108].

Cord length increases proportionally with the gestational age. A long umbilical cord is considered if the length exceeds 70 cm and it is considered short if measures less than 30 cm [71]. Short umbilical cord can be associated with fetal inactivity, fetal malformations, myopathic and neuropathic diseases, oligohydramnios, and some syndromes [72]. Long cord is associated with placental lesions, fetal growth restriction, intrauterine hypoxia, and even fetal death; additionally, cord accidents, entanglement, knotting, and prolapse can lead to long-term adverse neurologic outcomes. Antenatal ultrasound assessment of umbilical cord length is extremely difficult and uncertain.

## 3. Proposed Classification

Our recommendation regarding umbilical cord anomaly classification includes two classes:

Class S of structural abnormalities of the umbilical cord, and Class P of positional abnormalities. Due to the fact that the localization of most anomalies influences perinatal outcome, aiming to simplify the reporting and increase the accuracy of umbilical cord anomaly diagnosis, we encoded fetal insertion, free umbilical cord and placental insertion (Table 2 and Table 3).

## 4. Discussions

This paper reviews the most important aspects of the structural abnormalities of the umbilical cord, as determined by prenatal ultrasound, highlighting their clinical relevance for the management of these high-risk pregnancies.

Beginning with the normal structure of the umbilical cord, with two arteries branching off the left and right internal iliac arteries, and one vein formed by the confluence of chorionic veins at the chorionic plate, the continuous, increasing incidence and update on the aspects of umbilical cord abnormalities make this subject an ever-topical one.

Compared with pregnancies without abnormalities of the umbilical cord, the presence of this pathology induces an increased risk of polyhydramnios, birth before 34 weeks, placental abnormalities and low birth weight; implicitly, there is an increased index of cesarean delivery due to the presence of fetal distress, increased prenatal mortality rate and higher admission to neonatal intensive care.

Current guidelines highlight the importance of determining the number of vessels and fetal cord insertion. However, lately, the focus has been increasingly on the diagnosis of other abnormalities of the umbilical cord, especially in terms of their frequent association with numerous fetal abnormalities. The normal fetal and placental insertions of the umbilical cord and its structure should be documented after 12 weeks of gestation. Umbilical cord anomalies are usually seen clearly after 20 weeks using the standard two-dimensional technique in transverse and longitudinal planes; however, the image is improved by color Doppler and three-dimensional imaging techniques [109]. A more detailed examination includes the description of the fetal and placental insertion sites, the helical pattern of the umbilical arteries, and the characteristics of the Warton’s jelly [71]. The extended analysis of the umbilical cord offers the advantage of identifying and preventing adverse perinatal outcomes associated with certain umbilical cord abnormalities such as thinness, velamentous insertion, vasa praevia, abnormal coiling, cysts and tumors [110].

The obstetrician is faced with an ethical dilemma when observing an unusual coiling of the umbilical cord that may indicate a true umbilical cord knot—the “hanging noose” sign—upon ultrasound examination, without other specific symptoms and abnormal sonographic findings and normal Doppler assessment (when the measurements are performed in a free cord loop and the Doppler indices—pulsatility; resistance; and the peak-systolic/end-diastolic velocity ratio are within normal ranges [9]): the decision must be made to either inform the patient of the suspicion of a true umbilical cord knot and the risks related to this condition, and the decision to preventing patient’s anxiety and iatrogenic preterm birth by close and apparently unjustified monitoring. In fact, the debate could be resumed to positioning this pathology among clinically relevant UCA or among incidental findings. The decision belongs to International Societies that have to respond to the evident need of clarify the feasibility and strategies of the ultrasound antenatal scan for detection of the umbilical cord anomalies. Practically, HD flow Doppler should be used at the site of the coiling when an umbilical cord knot is suspected. If the technology or operator experience are not available at the screening site, suspected cases should be referred to fetal medicine units.

We propose a significant change in the practice of fetal ultrasound, which is likely to prove useful. A prospective study based on our proposed classification is the next step to be undertaken. Our article is written after 10 years of detailed and extensive assessment of the umbilical cord during the entire gestational period; looking for anomalies is the first step in discovering them, and the changed medical attitude may prevent, over time, many adverse pregnancy outcomes.

## 5. Conclusions

Although we acknowledge that some umbilical cord anomalies might develop later in pregnancy and may be obscured from view (about 0.3%), we consider that marginal or velamentous cord insertion should be routinely performed at the first and second trimester ultrasound screening for placental umbilical cord insertion, and trans-vaginal ultrasound color-Doppler assessment of internal cervical os—during the mid-trimester scan, at the time of cervical length measuring—when screening for premature delivery, at least in cases of low-lying placentas. Umbilical cord insertion should be assessed starting with the first trimester and continuing into the second trimester. Even if the incidence of some of the umbilical cord abnormalities is low, the fact that many of these are life threatening—and the cause of prenatal and intrapartum fetal morbidity and mortality (vasa praevia, cord knot)—we consider that it is mandatory to introduce this in the evaluation guidelines of both cord insertions, and scanning of the cord between the insertion sites during the ultrasound second trimester screening for fetal abnormalities; from our experience, most knots are formed up to this gestational age. The amount of amniotic fluid in the second trimester allows easy depiction of cord anomalies.

## Figures and Tables

**Table 2:** Proposed classification of the Umbilical Cord Anomalies.

Proposed UCA Class	Clinical Useful UCA	Incidental Finding of UCA	Clinical Un-Useful UCA
Class S	Velamentous Cord Insertion	Cord Tumors	Right Umbilical Vein Persistence
Class S	Vasa praevia	Umbilical Cord Cyst	Isolated Cord Varix
Class S	Omphalocele	Cord Hematoma	Excessive/Absent Coiling
Class S	Single Umbilical Artery	Cord Strictures	Abnormally short/long Cord
Class P	Cord Knot	Funic Cord Presentation	Nuchal Cord < 3 loops
Class P		Eccentric/Marginal Cord Insertion	

**Table 3:** Management of the UCA classified by clinically usefulness.

	Type of Anormaly	Management
Clinically Useful	Velamentous Cord Insertion	Fetal anatomic survey, serial assessment of fetal growth every 4 to 6 weeks
	Vasa praevia	Administration of corticosteroids at 28–32 weeks for accelerate pulmonary maturation Hospitalization at 30–32 weeks in tertiary care unit Elective caesarean section prior to membrane rupture, at 34–36 weeks Aggressive resuscitation of the neonate in case of ruptured vasa praevia
	Omphalocele	Karyotyping (amniocentesis), therapeutic abortion or expectant management with fetal anatomic survey and serial assessment of fetal growth
	Single Umbilical Artery/Umbilical Artery Hypoplasia	Detailed fetal anatomical survey, assessment of the placenta and umbilical cord; cell-free DNA screening for isolate SUA/invasive karyotype with microarray for non-isolated SUA; monitor for growth restriction with Doppler velocimetry assessment of the single/larger diameter umbilical artery
	Cord Knot	Close fetal monitoring in the third trimester by serial nonstress tests, biophysical profile scoring and Doppler assessment Elective cesarean delivery at 38-weeks of gestation
Incidental finding	Cord Tumors	Detailed fetal anatomical survey and monitoring for partial occlusion of umbilical blood flow
	Umbilical Cord Cyst	Detailed fetal anatomical survey and monitoring cystic diameter, and possible obliteration of umbilical blood flow; invasive karyotype with microarray for non-isolated cysts; repeated fetal growth assessment in the third trimester.
	Cord Hematoma	Monitor for growth restriction and fetal distress particularly during labor Check for reduction in fetal movements Cesarean section is highly recommended
	Funic Cord Presentation	Carefully assess membrane rupture
	Eccentric/Marginal Cord Insertion	Monitor for growth restriction and fetal distress particularly during labor
	Cord Strictures	Monitor for growth restriction and fetal distress particularly during labor
Clinically Unuseful	Right Umbilical Vein Persistence	Careful examination of fetal anatomy and exclusion of conjoined twins in twin pregnancy
	Cord Varix	Frequent nonstress testing and ultrasound surveillance. Cases associated with IUGR should be delivered when fetal lung maturation is achieved, at 34–36 weeks
	Abnormal coiling and length of umbilical cord	Close fetal monitoring in the third trimester by serial nonstress tests, biophysical profile scoring and Doppler assessment
	Nuchal Cord	Carefully assessment of labor

## Supplementary Materials

The following are available online at https://www.mdpi.com/article/10.3390/diagnostics12020236/s1, Figure S1: scanning images of various nuchal cords observed in the clinical practice; Figure S2: three-dimensional imaging of varix cords; Figure S3: cystic abnormalities; Figure S4: coiling pathologies: lax cord; Video S1: NOD_26.
