# Supplementary material for: Clinically Relevant Prenatal Ultrasound Diagnosis of Umbilical Cord Pathology
Source: Diagnostics (Basel). 2022 Jan 19;12(2):236. doi: 10.3390/diagnostics12020236 (PMC8871173; doi:10.3390/diagnostics12020236)
Supplement: Supplementary file 1 [file diagnostics-12-00236-s001.zip › Supplementary Files-4-final2.pdf]

Clinically Relevant Prenatal Ultrasound Diagnosis of Umbilical Cord Pathology

Roxana Elena Bohîlțea [1,2,*], Vlad Dima [2,*], Ioniță Ducu [3], Ana Maria Iordache [4,*], Bianca Margareta Mihai [2], Octavian Munteanu [5], Corina Grigoriu [1,3,*], Alina Veduță [2], Dimitrie Pelinescu-Onciul [1] and Radu Vlădăreanu [1]

[1] Department of Obstetrics and Gynecology, "Carol Davila" University of Medicine and Pharmacy Bucharest, 37 Dionisie Lupu, 020021 Bucharest, Romania; dimitriepelinescu@yahoo.com (D.P.-O.); vladareanu@gmail.com (R.V.)

[2] Department of Obstetrics, Gynecology and Neonatology, Filantropia Hospital, 11–13 Ion Mihalache Blv., Sector 1, 011171 Bucharest, Romania; bmmihai@gmail.com (B.M.M.); alina.veduta@gmail.com (A.V.)

[3] Department of Obstetrics and Gynecology, University Emergency Hospital, 169 Splaiul Independentei Bld., sector 5, 050098 Bucharest, Romania; ionitaducu@gmail.com

[4] Optospintronics Department, National Institute for Research and Development in Optoelectronics-INOE 2000, 409 Atomistilor, 077125 Magurele, Romania

[5] Department of Anatomy, "Carol Davila" University of Medicine and Pharmacy Bucharest, 37 Dionisie Lupu, 020021 Bucharest, Romania; octav_munteanu@yahoo.com

* Correspondence: r.bohiltea@yahoo.com (R.E.B.); dima.vlad@yahoo.com (V.D.); ana.iordache@inoe.ro (A.M.I.); corigri@gmail.com (C.G.)

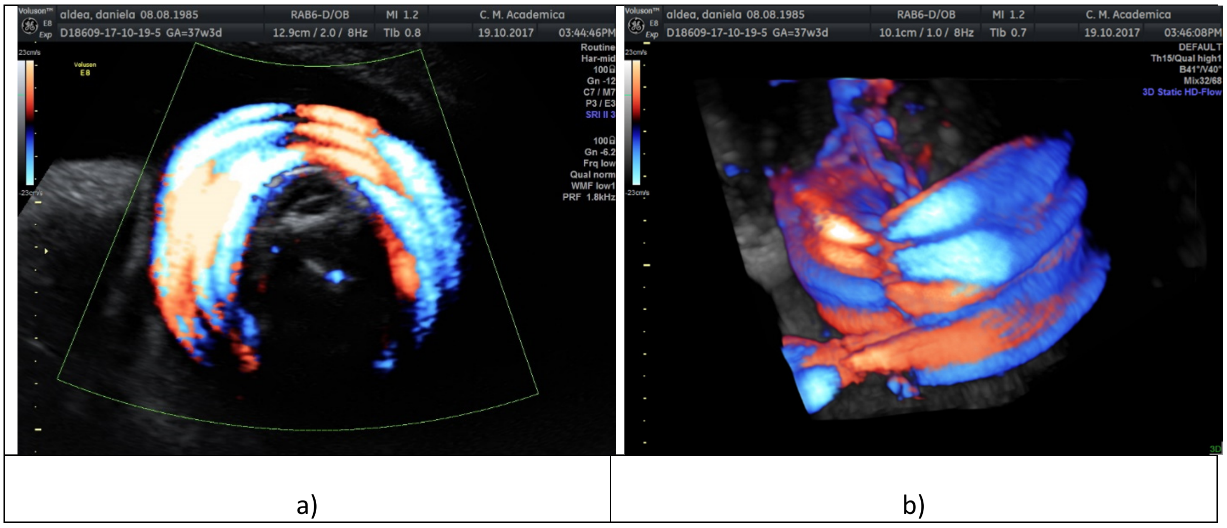

a)	b)

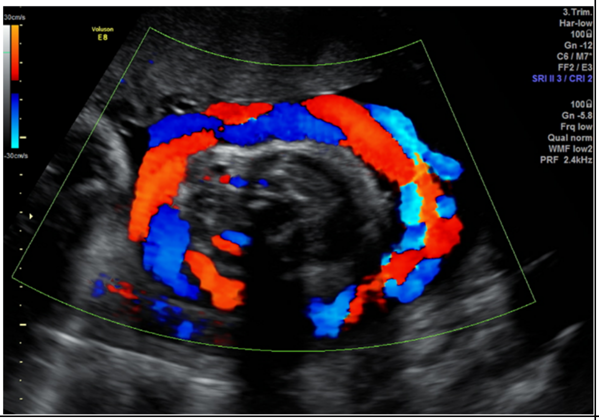

c)

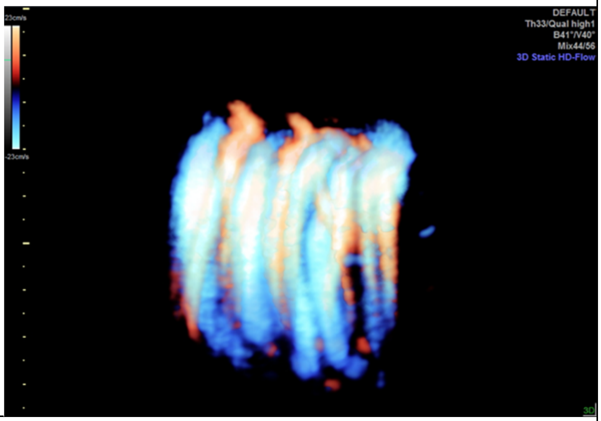

d)

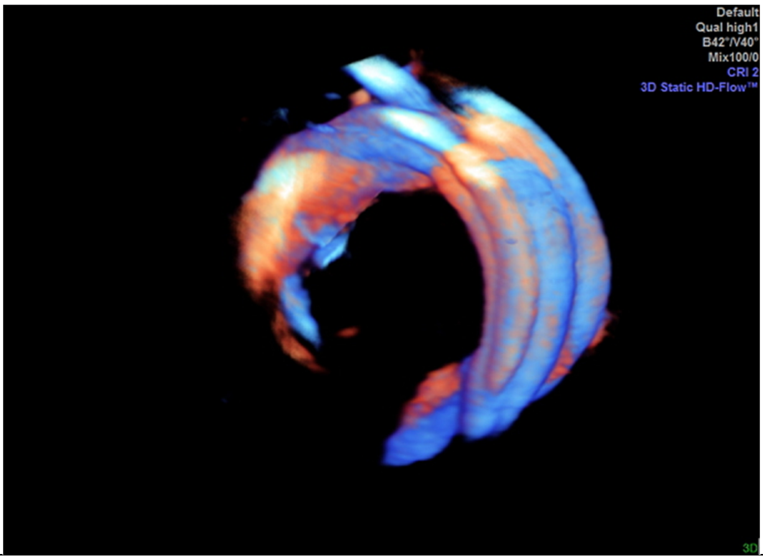

e)

Figure S1. Scanning images of various nuchal cords observed in the clinical practice

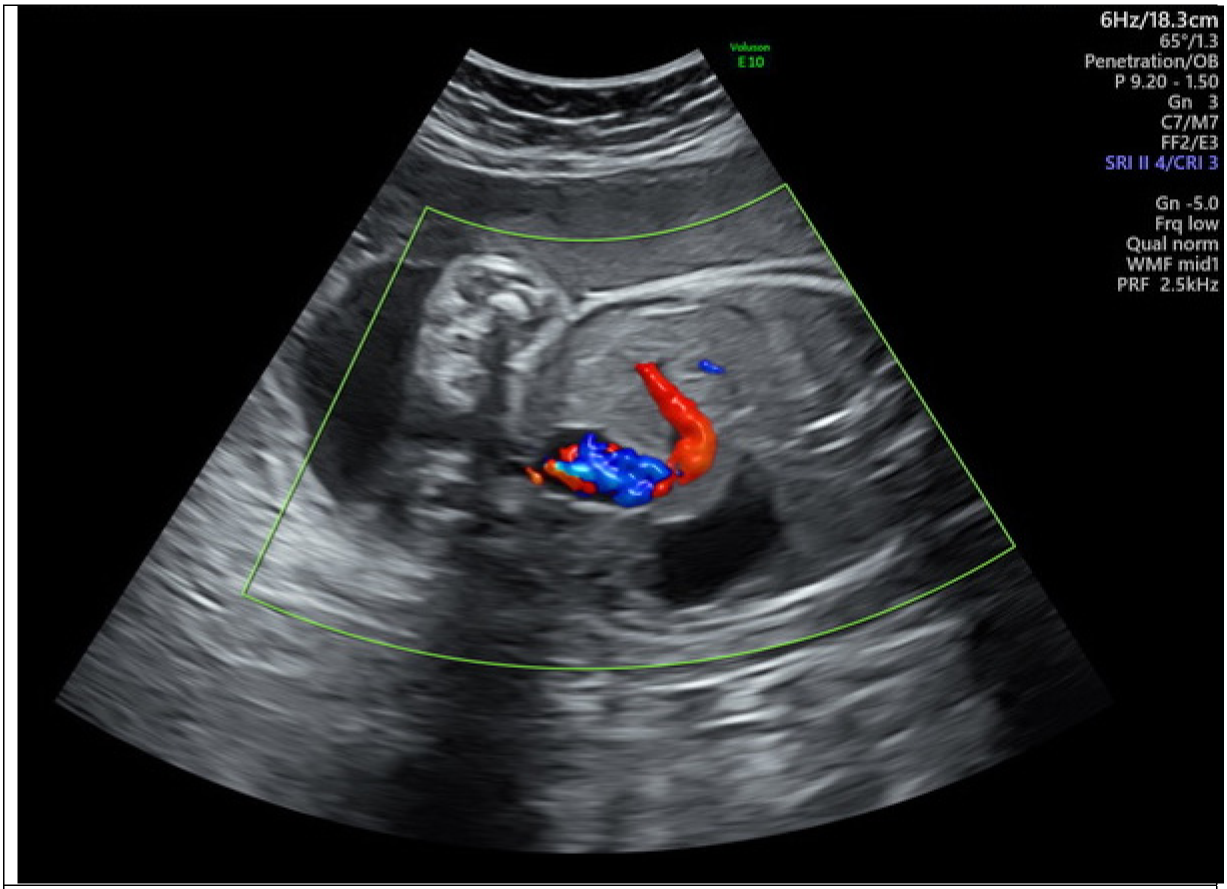

Figure S2. 3D imaging of varix cords

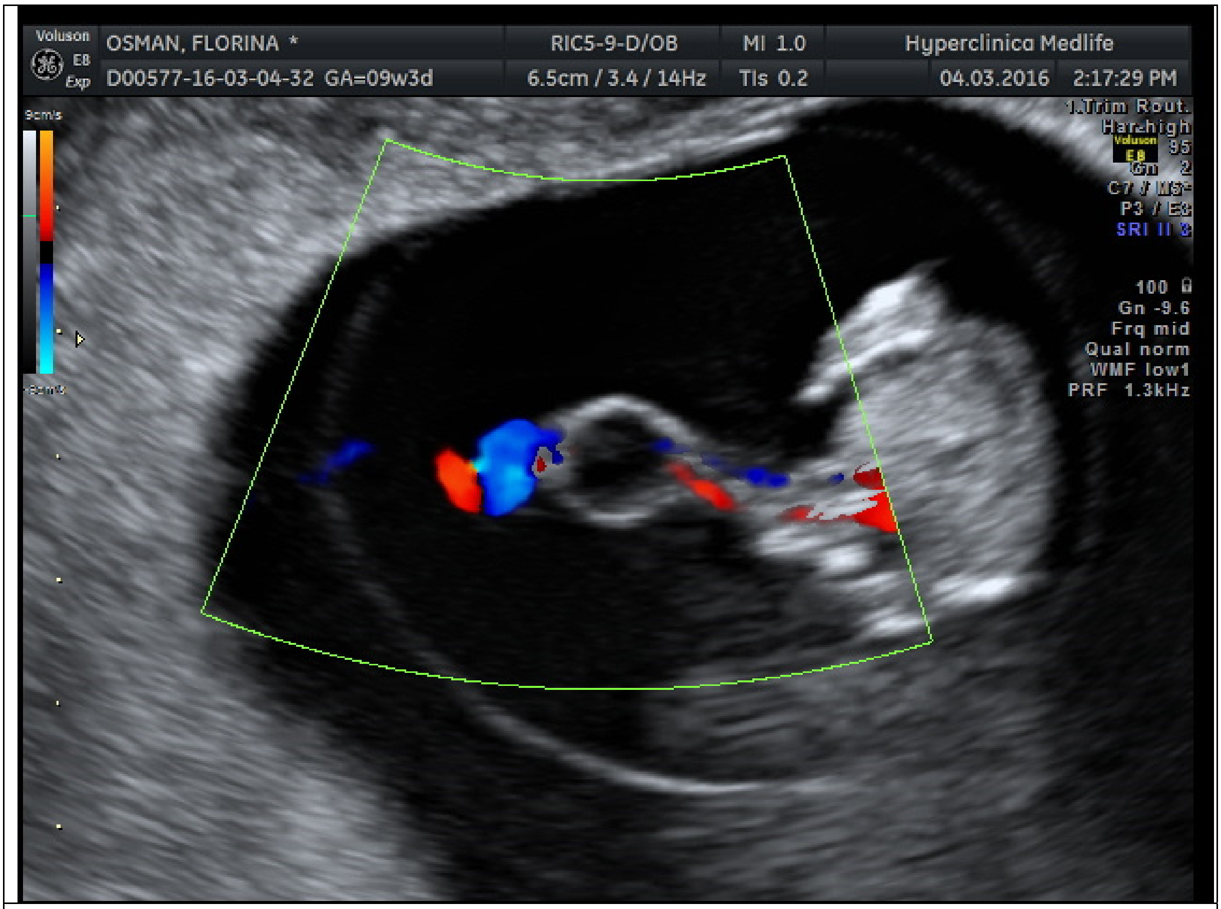

Figure S3. Cystic abnormalities

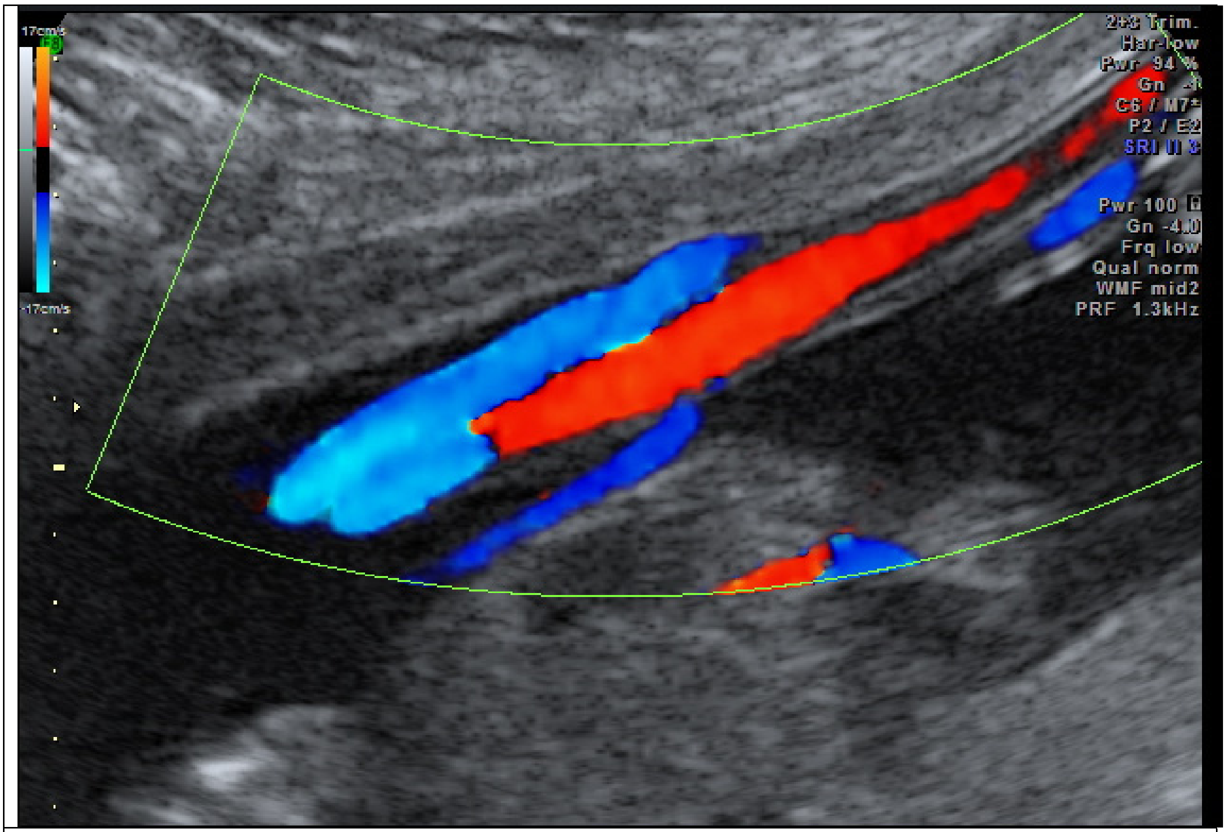

Figure S4. Coiling pathologies: lax cord
